# Measles burden in urban settings: characteristics of measles cases in Addis Ababa city administration, Ethiopia, 2004-2014

**DOI:** 10.11604/pamj.supp.2017.27.2.10677

**Published:** 2017-06-09

**Authors:** Amare Mengistu Mersha, Fiona Braka, Kathleen Gallagher, Aysheshim Ademe Tegegne, Aron Kassahun Argay, Mekonnen Admassu Mekonnen, Merawi Aragaw, Debritu Mengesha Abegaz, Etsehiwot Zeamlak Worku, Mekonen Getahun Baynesagn

**Affiliations:** 1 World Health Organization, Ethiopia; 2 Federal Democratic Republic of Ethiopia, Ministry of Health; 3 Addis Ababa City Administration health Bureau; 4 Federal Democratic Republic of Ethiopian, Ethiopian Public Health Institute

**Keywords:** Measles, surveillance, mortality, elimination, outbreak

## Abstract

**Introduction:**

In developing countries, measles was a major cause of morbidity and mortality before the wide spread use of measles vaccine. The purpose of this study was to describe measles burden in an urban setting, Addis Ababa- since the implementation of measles case-based surveillance in Ethiopia. We analyzed measles surveillance data for 2004 -2014.

**Methods:**

Incidence of measles was described by sub city, by year and by age groups. Age specific incidence rate were calculated. Logistic regression was used to identify the predictors of confirmed measles cases.

**Results:**

Of 4220 suspected measles cases 39% were confirmed cases. Males and females were equally affected. The mean affected age was 7.59 years. Measles cases peaked in 2010 and 2013-2014. Incidence of measles is higher among children less than five years old. Outer sub cities were more affected by measles in all years.

**Conclusion:**

Sub cities bordering with Oromia Regional State were more affected by measles. Older age groups were more affected than younger age groups (age ≤ five years old). Efforts to close immunity gaps against measles and further strengthen surveillance in urban settings, particularly among children below five years old, should be prioritized.

## Introduction

Measles is an acute viral illness with the potential for severe and life threatening complications. In the pre-vaccine era, measles was one of the major causes of childhood morbidity and mortality. Although significant reduction in morbidity and mortality has been achieved since the widespread use of measles vaccine, it is still a major global public health problem [1]. Measles incidence is associated with urbanization because of high influx of migrants and high population density; cities have become important hubs for the transmission of infectious diseases [2–10]. In 2000, a total of 39.9 million measles cases, and 777,000 deaths were reported globally; Africa and Southeast Asia accounted for 70% of measles cases and 84% of measles-related deaths. Eleven countries including Ethiopia, accounted for 66% of deaths [11]. In 2001, the African Region adopted the Global Measles Mortality Reduction Initiatives and began the implementation of the recommended strategies. The region set the following targets to be achieved by the year 2012: 1) to improve measles containing vaccine (MCV1) coverage. MCV1 is the first dose of measles containing vaccine, 2) to provide a second opportunity for measles vaccine (MCV2), MCV2 is the second dose of measles containing vaccine 3) to improve measles case management, and 4) to establish case-based surveillance with laboratory confirmation of all suspected measles cases. Successful implementation of these strategies resulted in a 93% reduction in reported measles cases and 92% reduction in the estimated number of measles deaths in the African region between 2000 and 2008 [12–14]

In 2008, the African Region, adopted a pre-elimination goal to be reached by the end of 2012 with the following targets: 1) greater than 98% reduction in estimated regional measles mortality compared with 2000; 2) annual measles incidence of fewer than five reported cases per million population nationally; 3) greater than 90% coverage national and >80% MCV1 districts, and 4) greater than 95% MCV coverage in all districts during supplementary immunization activities (SIA) [15] Ethiopia failed to achieve most of the targets for the pre-elimination goal in 2011:- the estimated MCV1 coverage was 57% in 2011 and the percentage of districts reporting at least ≥ 80% MCV1 coverage was 43 % in 2011 (16). However, the administrative coverage of the 2010-2011 nationwide follow up measles SIAs in children under 5 years of age was 106 % with 91% of districts achieving at least 95 % coverage. National coverage for the SIA based on a population survey was 88.2 % (95% CI=85.1%, 90.6%) In 2011, reported measles cases among children under 5 years of age decreased by 66% compared with 2010 [16] In 2014, data from the Addis Ababa City Administration Health Bureau Health Information and Management System (HIMS) indicated that all sub cities in the city administration reported at least one measles outbreak, with a total of 276 suspected measles cases.

We reviewed 4220 measles records to describe measles incidence in Addis Ababa between 2004 and 2014, in effect reviewing 10 years since the implementation of cases based measles surveillance in Ethiopia. We also reviewed MCV1 coverage from 2005 to 2013 and tried to assess the impact of measles vaccination among under 5 years old children (improvement in routine immunization coverage over years is associated with declining in the number of measles cases in children under 5 years of age). The study was conducted to propose effective interventions for measles elimination to the city administrators.

## Methods

**Study area and setting:** Addis Ababa is the capital city of Ethiopia with an estimated 2015 population of 3,194,999 [17]. The city is divided into 10 sub cities (which are equivalent to zones) and 116 woredas, which are equivalent to districts [17]. There are five central, six regional and 36 private hospitals, 93 public health centers and more than 500 private health facilities. In Addis Ababa, surveillance data for suspected measles cases are routinely collected at all levels of the health delivery system using standardized measles case based surveillance form and specimens are sent to the national measles laboratory. A single serum specimen is collected for serologic confirmation of measles at the first contact with the case any time between the day of onset of rash and 30th day afterwards. Data from the health facilities are sent to the national measles laboratory with the serum specimen, copied to sub cities, and the regional health bureau. Data is entered into the measles case-based surveillance system database, for consolidation, cleaning, analysis and dissemination for action. Monthly measles immunization performance reports are sent from health facilities to higher levels for analysis and action. Measles vaccination coverage was calculated by dividing the number of doses of measles vaccine given to children in the target age group by the number of surviving infants estimated by the Central Statistical Agency (CSA) [17] Important variables captured in the case-based surveillance forms include:- patient´s name, age, sex, vaccination status, area of residence, date of rash onset, date of first investigation, dates of specimen arrival and dispatch of results from the national virology laboratory, specimen condition on arrival at national virology laboratory, measles IgM test result, and final case classification. Feedback on the final case classification is given to the reporting facility.

**Study design:** we conducted a retrospective record review of the national measles- surveillance dataset for the period of 2004 - 2014 and measles coverage data for the period of 2005 - 2013 to assess the incidence of measles cases by sub city, year, and age group and calculate age specific incidence rate by year. Measles vaccination data was not found for the year 2004. Age specific population data from 2004-2014 were used to calculate incidence rates [17].

**Data analysis:** we assessed achievement of the city administration towards the target set for measles mortality reduction and pre-elimination goals. The proportion of confirmed measles cases were calculated from total reported cases. Logistic regression was done to assess the association between the dependent variable (measles cases) and the independent variables (sex, age, and timing of specimen collection). Permission and ethical clearance was obtained from Ethiopian Public Health Institute; Patient names and addresses were omitted from the analysis. Confidentiality was assured and maintained.

### Definitions

**Suspected measles case:** any person with fever and generalized rash and cough and runny nose or conjunctivitis.

**Confirmed measles case:** a measles IgM positive case or a suspected measles case linked to a laboratory confirmed cases or a compatible case.

**Compatible measles case:** a suspected case with no laboratory testing or with measles IgM test that is repeatedly indeterminate.

**Measles outbreak:** five or more suspected measles cases per month per district.

## Results

**Figure 1:**
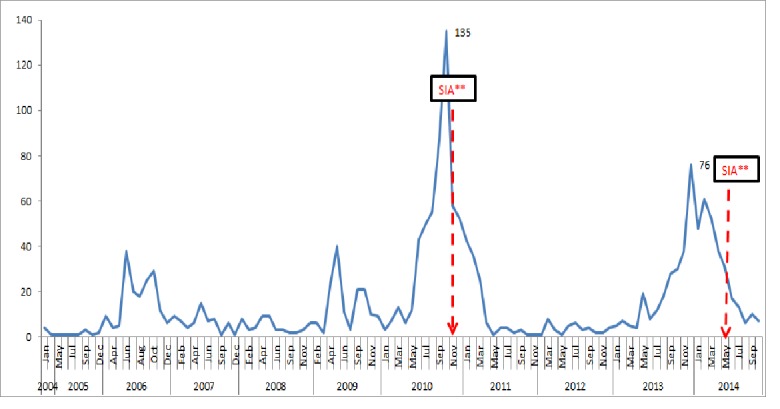
Trends of main surveillance performance indicators 2005-2015

We reviewed a total of 4220 measles records of which: 1659 (39%) were confirmed measles cases. The female: male ratio was 1:1. The mean age of the suspected measles cases was 7.59 years with standard deviation (+SD) of 6.96 years. Measles cases were reported throughout 2004-2014: two small peaks were noted in October 2006 and 2009. In 2010, 521 confirmed cases of measles were reported before the measles SIA, a total of 250 and 283 confirmed measles were reported in 2013 and 2014 respectively (Figure 1).

The incidence rate of measles cases per 100,000 populations per year was higher in age groups between 1 and 5 years (range 13 to 125 cases/100,000), followed by children under 1 year of age, children 5-15 years and persons older than 15. In 2010, the annual incidence rate of confirmed measles cases was 18.1/100, 000 population per year, in 2013 measles incidence rate was 8 cases /100,000 population per year and in 2014 the incidence rate was 8.9 cases/100,000 population per year (Table 1). Regional measles vaccination coverage was 81%, 79% and 92% in 2009, 2010 and 2011 respectively. In 2013, the regional coverage was 95%: ranging from 58 % in Gulele and to 122% in Bole sub city (Table 2). The lowest number of measles cases were reported in 2004 (5), 2005 (10) and 2012 (39). Peaks in confirmed measles cases were observed in 2009-2010 and 2013-2014, with the highest number recorded in 2010 with 521 cases (Table 3). Confirmed measles cases were reported by 20% of the sub cities in 2004 and by 40 % of the sub cities of the Addis Ababa city administration in 2005. There was no confirmed measles case reported by Akaki Kality sub city in 2011 and by Lideta sub city in 2012. Large numbers of measles cases were reported by all sub cities in 2010, 2013 and 2014 (Table 3).

**Table 1:** Incidence rate confirmed measles cases per 100, 000 population per year by age group, 2004-2014, Addis Ababa, Ethiopia

Year	Age group (years)			
	<1	1-5	5-15	>15	Overall incidence rate
**2004**	13	13	3	0	0.2
**2005**	16	54	12	0.3	0.4
**2006**	84	120	25	3.4	6.4
**2007**	44	53	11	1.3	2.4
**2008**	35	76	14	1	1.9
**2009**	37	55	21	1.3	5.2
**2010**	208	146	45	7.2	18.1
**2011**	100	67	17	2.5	4.2
**2012**	43	142	45	2	1.3
**2013**	95	122	44	5.5	8.0
**2014**	122	125	50	5.2	8.9

**Table 2:** Administrative coverage of measles vaccination through routine services by sub city and region wide, 2005–2013, Ethiopia Region

Sub city	2005	2006	2007	2008	2009	2010	2011	2012	2013
Addis Ketema	35	35	37	46	64	73	72	52	60
Akaki Kality	65	53	57	59	58	63	104	113	104
Arada	69	57	57	70	111	112	123	117	120
Bole	70	66	71	94	100	91	122	119	122
Gulele	46	54	40	44	48	42	71	58	58
Kirkos	50	44	41	46	76	61	71	99	76
Kolfe Keranio	85	88	81	93	69	107	101	119	116
Lideta	94	73	79	79	130	89	77	73	73
Nefas-Silk Lafto	84	82	77	87	98	69	104	104	102
Yeka	90	81	51	47	68	71	73	83	76
**Total**	**68**	**63**	**59**	**67**	**81**	**79**	**92**	**95**	**92**

**Table 3:** Distribution of confirmed measles cases by sub city by year, 2004- 2014, Addis Ababa, Ethiopia

Addis Ketema	Akaki Kality	Arada	Bole	Gulele	Kirkos	Kolfe Keraniyo	Lideta	Nefas Silk L	Yeka	Total
0	0	1	0	0	0	0	4	0	0	5
0	1	5	2	0	2	0	0	0	0	10
15	1	13	35	14	14	36	13	15	10	166
1	24	8	9	2	2	10	1	2	5	64
7	2	9	13	4	2	2	2	7	4	52
4	33	9	5	10	23	30	5	13	13	145
46	24	42	51	60	56	101	48	53	40	521
10	0	14	8	25	6	21	11	12	18	125
3	1	4	3	6	2	12	0	3	5	39
18	38	14	38	22	9	45	17	21	28	250
19	12	13	35	36	13	62	15	38	40	283

**Figure 2:**
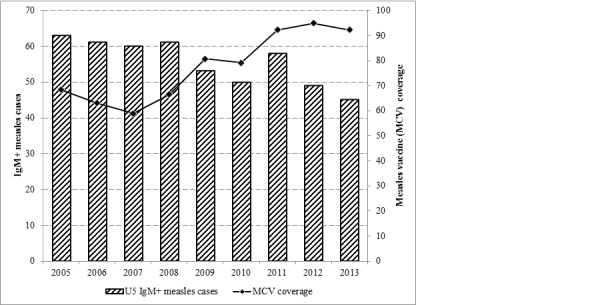
Proportion of under 5 years’ old IgM+ measles cases, city level measles (MCV1) administrative coverage, Addis Ababa, Ethiopia, 2005–2013

In 2005, 63% of IgM+ measles cases were children age below 5 years old, where as in 2013 the proportion reduced by 18% compared to 2005; of all IgM+ measles cases 45% were age below 5 years old. An eight percent increase in the proportion of less than five years old confirmed measles cases was noted in in 2011 compared to 2010. The regional measles vaccination coverage was 68 % in 2005 and 92% in 2013 (Figure 2). In 2004 and 2008, the proportion of confirmed measles cases in children under 5 years old was higher compared with the proportion of confirmed cases among above 5 years old. However, in 2013, the proportion of confirmed measles cases in children under 5 years old age group was 10%, lower than the proportion of confirmed measles cases in above 5 years old age group ( 28 % in 2013) (Table 4).

**Table 4:** Distribution of confirmed measles cases in less than five and above five years by year

Year	< 5years		≥ 5 years	
	No	%*	No	%*
2004	3	8	2	5
2005	3	2	7	5
2006	73	17	93	22
2007	27	15	37	20
2008	31	14	21	9
2009	60	25	85	35
2010	240	32	281	38
2011	56	18	69	22
2012	17	3	22	4
2013	65	10	185	28
2014	85	12	198	28

* % calculated from the total (confirmed cases cases) + discarded

Logistic regression analysis showed that female suspected measles cases were 1.4 times more like to be confirmed than males (95% CI= 1.18-1.58). Children older than 5 years were 1.5 times more likely to be confirmed measles cases compared with those less than 5 years old (95 % CI= 1.30-1.74). Suspected measles cases from whom serum specimen was collected after ≥4 or more days were 2.4 times more likely (95% CI =2.02-2.73)to be confirmed measles cases compared with cases in whom specimen was collected within 4 days of rash onset.

## Discussion

In our review of measles surveillance data from Addis Ababa in the years 2004 to 2014, we found that sub cities in Addis Ababa which share a common border with Oromia regional state reported more measles cases. All of the six outer sub cities are expansion and resettlement areas. Influx of migrants from rural areas could have led to overcrowding and created favorable conditions for measles transmission. The relationship between urbanization and measles control has shown that measles is a common cause of childhood morbidity in peri-urban areas which could be related to high population density, and low measles vaccination coverage [8]. Surprisingly relatively fewer confirmed measles cases were reported by Akaki Kality sub city in 2006, 2010, and 2014 compared with inner sub cities. Akaki Kality sub city is one of the expansion areas, shares a common border with Oromia regional state and with relatively low health service coverage and low measles vaccination coverage. The sub city was expected to report more measles cases than the inner sub cities.

In 2004-2005, during the first 2 years of case based measles surveillance implementation, only 15 cases of confirmed measles cases were reported. The low measles reporting rate could have been due to lack of awareness of the new measles case based measles surveillance system. Significant progress was made in measles surveillance activities starting from 2006: all sub cities have been reporting at least one measles case per year. Improvement in measles performance indicators were also recorded at the regional level with several efforts made to strengthen surveillance through capacity building among other interventions [12].

Our study found that the incidence rate of confirmed measles cases per 100,000 people was highest among children aged 1-5 years in all years except in 2010 and 2011 when it was highest among under 1 year olds; incidence was lowest in the age group above 15 years old. This was comparable to findings from a study in New Zealand which reviewed measles cases from three sources:- hospitalization, notification and laboratories [18]. We observed a declining trend in measles cases in less than 5 years old age group compared to the above 15 years old age group. This could be explained by improved routine immunization coverage in general and measles vaccination coverage in particular in more recent years [18]. Large numbers of measles cases were reported in 2014, six months before the nationwide 2013 SIA. Of 446 reported measles cases 189 (32 %) were aged between 1 and 4 years. These children were the target age group for measles SIAs in 2013, implying poor quality planning of the 2013 measles SIA in Addis Ababa.

We recognize some limitations of our study. Firstly, the denominators that we used to calculate, incidence and vaccine coverage may be inaccurate due to high urban- rural migration. This could tend to increase the incidence rate. Reported measles cases (numerator) were not also included in the denominator. Secondly, there may be under reporting of measles cases for various reasons; measles cases may not be allowed to get out of the home and are frequently managed at home with diet, herbal drinks and bath [19]. Hence, a significant number of parents may not seek medical assistance for their sick children, believing measles is self-limiting diseases. However, we do not feel that either of these limitations significantly affected the findings of our study.

## Conclusion

In conclusion, our study showed that measles is still a common cause of childhood morbidity, and Addis Ababa sub cities bordering the Oromia Region were the most affected. We also conclude that improvement in routine immunization coverage over years is associated with declining in the number of measles cases in children under 5 years of age. We recommend that government and partners involved in the immunization program revisit their approach in addressing measles pre-elimination goals through: - strengthening routine immunization, conducting higher quality SIAs and achieving measles surveillance goals in all sub cities of Addis Ababa. We recommend that surveys be done in the outer sub cities of Addis Ababa to identify high risk areas for measles transmission in view of limitations in accurate population estimate in the city administration given population migration. We also recommend further study to assess the effect of urbanization on measles morbidity (the effects of overcrowding and influx people from rural areas where vaccination coverage is suboptimal on measles burden). We also recommend that the city administration regional health bureau, the six outer sub cities health offices, and neighboring woredas should closely work with other sectors offices and the Oromia Regional Bureau.

### What is known about this topic

- Measles account for much of the vaccine preventable diseases burden in Ethiopia;
- Measles is the major contributor for less than five years old morbidity and mortality in Ethiopia;
- Measles incidence has been declining since the introduction of measles vaccine in Ethiopia.

### What this study adds

- The study provides valuable information on measles distribution in urban settings: Addis Ababa;
- The study provides information on the impact of vaccination on measles incidence in Addis Ababa;
- The study also reveals that, the quality of measles supplementary immunization activities.

## Competing interests

The authors declare no competing interests. The views expressed in the perspective articles are those of the authors alone and do not necessarily represent the views, decisions or policies of the institutions with which they are affiliated and the position of World Health Organization.
